# Chronic Hyperglycemia Induces Trans-Differentiation of Human Pancreatic Stellate Cells and Enhances the Malignant Molecular Communication with Human Pancreatic Cancer Cells

**DOI:** 10.1371/journal.pone.0128059

**Published:** 2015-05-26

**Authors:** Katalin Kiss, Kornélia Baghy, Sándor Spisák, Szilárd Szanyi, Zsolt Tulassay, Attila Zalatnai, J.-Matthias Löhr, Ralf Jesenofsky, Ilona Kovalszky, Gábor Firneisz

**Affiliations:** 1 1st Department of Pathology and Experimental Cancer Research, Semmelweis University, Budapest, Hungary; 2 Children's Hospital, Harvard Medical School, Boston, MA, United States of America; 3 Department of General Surgery, University of Heidelberg, Heidelberg, Germany; 4 School of Ph.D. Studies, Semmelweis University, Budapest, Hungary; 5 2nd Department of Internal Medicine, Semmelweis University, Budapest, Hungary; 6 Karolinska Institutet, Gastrocentrum, Karolinska University Hospital, Stockholm, Sweden; 7 University of Heidelberg, Medical Campus Mannheim, Dept. of Medicine II, Mannheim, Germany; University of South Alabama Mitchell Cancer Institute, UNITED STATES

## Abstract

**Background:**

Diabetes mellitus is linked to pancreatic cancer. We hypothesized a role for pancreatic stellate cells (PSC) in the hyperglycemia induced deterioration of pancreatic cancer and therefore studied two human cell lines (RLT-PSC, T3M4) in hyperglycemic environment.

**Methodology/Principal Findings:**

The effect of chronic hyperglycemia (CHG) on PSCs was studied using mRNA expression array with real-time PCR validation and bioinformatic pathway analysis, and confirmatory protein studies. The stress fiber formation (IC: αSMA) indicated that PSCs tend to transdifferentiate to a myofibroblast-like state after exposure to CHG. The phosphorylation of p38 and ERK1/2 was increased with a consecutive upregulation of CDC25, SP1, cFOS and p21, and with downregulation of PPARγ after PSCs were exposed to chronic hyperglycemia. CXCL12 levels increased significantly in PSC supernatant after CHG exposure independently from TGF-β1 treatment (3.09-fold with a 2.73-fold without TGF-β1, p<0.05). The upregualtion of the SP1 transcription factor in PSCs after CHG exposure may be implicated in the increased CXCL12 and IGFBP2 production. In cancer cells, hyperglycemia induced an increased expression of CXCR4, a CXCL12 receptor that was also induced by PSC’s conditioned medium. The receptor-ligand interaction increased the phosphorylation of ERK1/2 and p38 resulting in activation of MAP kinase pathway, one of the most powerful stimuli for cell proliferation. Certainly, conditioned medium of PSC increased pancreatic cancer cell proliferation and this effect could be partially inhibited by a CXCR4 inhibitor. As the PSC conditioned medium (normal glucose concentration) increased the ERK1/2 and p38 phosphorylation, we concluded that PSCs produce other factor(s) that influence(s) pancreatic cancer behaviour.

**Conclusions:**

Hyperglycemia induces increased CXCL12 production by the PSCs, and its receptor, CXCR4 on cancer cells. The ligand-receptor interaction activates MAP kinase signaling that causes increased cancer cell proliferation and migration.

## Introduction

Epidemiologic studies and their meta-analyses established a clear evidence for the association between diabetes mellitus (DM) and pancreatic cancer (PaC) and concluded that DM is not only an early manifestation, but also an etiologic factor of PaC.[1] Carstensen and co-workers based on the data of more than 4 million person-years confirmed the association between type 1 DM (T1DM) and PaC and concluded that a major carcinogenic effect of exogenous insulin is unlikely in T1DM. [2]. In the more prevalent type 2 DM (T2DM) the association with PaC is also evident in the view of a meta-analysis of 36 studies [3].

A prospective cohort reported that elevated fasting plasma glucose (FPG) levels are risk factors for PaC [4]. In addition, a dose-response meta-analysis of data obtained from 2408 PaC patients confirmed that every single mmol/L increase in FPG already above 4.1 mmol/L is associated with a 25% increase in the rate of pancreatic cancer [5]. In a risk model to identify individuals at increased risk for pancreatic cancer, diabetes >3 years posed a similar degree of risk than, family history of pancreatic cancer in the general population [6]. Pancreatic cancer, of which 90% of cases are ductal adenocarcinoma, means a miserable prognosis with a 5 years survival of 7% [7]. This means a uniquely high need for a better understanding of its molecular pathology.

Despite the number of supporting epidemiologic studies the cellular and molecular mechanisms for the evolution of this association between DM and PaC is less clear-cut. Therefore we hypothesized that chronic hyperglycemia in addition to the direct effect on cancer cells may also unfavourably alter the communication between cancer cells and the microenvironment, especially with the pancreatic stellate cells that are the major cellular stromal elements in PaC. The consequences of fibroblast activation—a process driven originally by transforming growth factor beta (TGF-β) evolved to enhance wound healing—are unfavorable in cancer disease [8]. Experimental data suggested that antitumor immunity was suppressed by stromal cells expressing fibroblast activation protein (FAP) and an agent targeting FAP-expressing cells enhanced anti-tumoral immunity [9]. However, this concept has been recently challenged based on observations with αSMA+ myofibroblast deleted transgenic mice in pancreatic cancer model suggesting an even more complex regulation [10].

Pancreatic stellate cells (PSCs) upon activation trans-differentiate to myofibroblasts-like cells that are the major source of the extracellular matrix (ECM) protein deposition during tissue fibrosis [11–13]. Pancreatic ductal adenocarcinoma is characterized by an abundant desmoplastic stroma as a result of PSC activation [14].

There is an intense communication between the tumor-associated PSCs and cancer cells. Activated PSCs release a variety of growth factors, cytokines and chemokines that promote the malignant behavior of pancreatic tumor cells, playing an essential role in cancer development and growth [15–18] Activated setllate cells also protect the pancreatic tumor from radiation—and gemcitabine—induced apoptotic effects [19]. PSCs in indirect co-culture enhanced stem cell-like phenotypes of cancer cells and induced the expression of cancer stem cell-related genes, suggesting a role in cancer stem cell niche [20].

We assessed the response of human PSCs exposed to chronic hyperglycemia (CHG, 21 days), using a multi-step approach to identify the molecular pathways that may regulate PSC activation. PaC cells were also directly assessed upon exposure to hyperglycemia or to conditioned PSC culture medium (CCM).

## Materials and Methods

### Cell lines, cell cultures

In all experiments we used the RTL-PSC and T3M4 human cell lines. RLT-PSC is a human pancreatic stellate cell line that was previously immortalized by transfection with SV40 large T antigen and human telomerase (hTERT) and analyzed for stellate cell markers [13]. In addition the T3M4 human pancreatic ductal adenocarcinoma cell line was used which was a kind gift from the European Pancreas Center in Heidelberg [21].

PSC cells were cultured in DMEM (Dulbecco's Modified Eagle Medium, Sigma, St. Louis, MO) with 1000 mg/L (5.5 mmol/L) glucose concentration, T3M4 cells were cultured in RPMI-1640 medium (Sigma) both supplemented with 10% fetal bovine serum (FBS, Sigma) and 1% Penicillin/Streptomycin (Sigma). Cells were cultured at 37 °C atmosphere containing 5% CO2 and passaged at 85–90% confluence using trypsin-EDTA solution (Sigma).

### Growth factors and other compounds

Transforming growth factor-β1 (TGF-β1, Sigma) was added in 5 ng/mL final concentration to the cells. AMD3100 octahydrochloride hydrate (2 μg/mL, Sigma) was used to inhibit CXCR4 receptor. During cell migration assessment cells were treated with Mitomycin C (10 μg/mL, Sigma, Part No.: M4287) to prevent cell proliferation. BSA (bovine serum albumin, Sigma, Part No.: A3294) was used for aspecific blocking in several methods.

### Treatment schedule of PSC and T3M4 cells

PSCs were exposed to CHG and TGF-β1 according to the following protocol:

In “control” conditions cells were cultured as described above. In case of “high glucose” treatment cells were cultured in culture medium containing 15.3 mmol/L glucose for 3 weeks (21 days), as preliminary experiments showed the best response of ECM protein production at this time-frame. Subsequently, cells were starved for 24 hours in FBS-free medium and thereafter for 48 hours in FBS-free medium either with or without TGFβ1 to compare its effect to CHG. Two biological parallels were used for each regimen.

Cells grown in T75 flasks were collected and used for mRNA and protein analysis, cell culture medium was collected for protein analysis. For immunocytochemistry the cells were grown on coverslips in 6 well plates. Experiments were repeated three times.

T3M4 cells were grown to 80% confluency in T75 flasks, then starved overnight in FBS-free RPMI. Subsequently culture medium, containing PSC-CCM and RPMI with 10% FBS in a 50%-50% ratio was added to the cells for 48 hours. As controls in the T3M4 experiments FBS-free DMEM (with 5.5 and 15.3 mM glucose concentration) was added in parallel to the full-FBS RPMI. After 48 hours of treatment, T3M4 cells and the cell culture supernatants were collected for protein studies.

### Preparation of conditioned cell culture medium (CCM)

We prepared CCM from RTL-PSCs by culturing them in T75 flasks with 12 mL DMEM containing 10% FBS for 3 days. DMEM with 1000 mg/L (5.5 mmol/L) or with 2750 mg/L (15.3 mml/L) glucose was used. CCM was collected from each flask, steril filtered, and stored at—20°C until use.

### mRNA expression array

Total RNA was isolated (Mean RNA Integrity Number [RIN] = 9.2 ± SD 0.4) using the RNeasy Kit (Qiagen, Hilden, Germany). Two biological duplicates were pooled within each group and two technical duplicates were hybridized from each pooled sample group onto the GeneChip PrimeView Human Gene Expression Array (Affymetrix, Santa Clara, CA). Biotinylated amplified RNA (aRNA) probes were synthesized from 200 ng total RNA using the 3’ IVT Express Kit (Affymetrix). Labeled aRNAs were then purified and fragmented for the subsequent hybridization. Fluorescent signals were scanned by GeneChip Scanner 3000 (Affymetrix). Data were extracted from the CEL files using „R” surface with Bioconductor software packages. Robust Multichip Average (RMA) normalization was performed and data were converted to Log2 notation to make Feature selection by linear model and SAM using „limma” and „samtools” packages. All microarray results are uploaded to Gene Expression Omnibus (GEO) database repository (accession number: GSE59953).

Gene expression values on each treatment arm were ranked upon their differential expression compared to the samples isolated from ‘control’ PSCs. Two sets of genes, the top 100 and 300 were selected to provide the best separation. (p-value: 10–4, Statistica software release 8, T-test).

### Bioinformatics/Pathway analysis

MetaCore (Thomson Reuters) pathway database integrated software was used for functional analysis of mRNA expression microarray data [22]. This analysis provided a preliminary rank of signal transduction pathways that are most likely altered in PSCs after CHG exposure. We have selected 14 differentially expressed genes from these orientational networks for further real-time RT PCR validation, based on their potential association with diabetes or contribution to PSC activation, islet specific fibrosis or pancreatic cancer.

### Real-time RT PCR validation

First strand cDNA was synthesized from 1 μg RNA using the M-MLV Reverse Transcriptase kit (Invitrogen by Life Technologies Carlsbad, CA, USA) under the conditions recommended by the manufacturer. Real-time PCR were performed using ABI Gene Expression TaqMan Assays (S1 Table.) and TaqMan Universal PCR Master Mix (Part No. 4324018) in an ABI 7000 Sequence Detection System, all purchased from Applied Biosystems by Life Technologies (Carlsbad, CA, USA) under conditions recommended by the manufacturer. Samples were run in triplicates in 20 μl total volume containing 50 ng cDNA [cycle conditions: denaturation: 95°C (10 min) followed by 40 cycles: 95°C (15s), annealing+extension: 60°C (1min)]. Results were standardized to 18S rRNA (Part No. 4319413E). Cycle threshold (CT) values were recorded and relative gene expressions were calculated using the 2^-ΔΔCT^ method.

### Enzyme-linked immunosorbent assay (ELISA)

Type-1 and type-3 collagen contents were assessed using an indirect ELISA system. Plates were coated overnight with cell culture supernatant at 4°C. After blocking with 3% w/v BSA, primary antibodies were used overnight at 4°C. After washing with PBS, appropriate secondary antibody was applied. (Antibody specifications and dilutions applied are indicated in S2 Table.) Signals were achieved by adding 3,3′,5,5′- tetramethylbenzidine (Sigma, Part No.: T0440), reaction was stopped by 1.6N sulfuric acid. Absorbance was recorded at 450 nm (Labsystems MultiScan MS, Thermo Labsystems, Milford, MA, USA).

Quantification of CXCL12 and IGFBP2 in cell supernatant was performed by using a Solid Phase Sandwich ELISA kit (Quantikine R&D Systems, Minneapolis, MN, USA, Cat. No.: DSA00 and DGB200), according to the manufacturer’s instructions. Each sample was tested in triplicates.

### Fluorescent immunostaining

For detection of intracellular type-1 collagen, alpha smooth muscle actin (α-SMA) and vimentin, immunocytochemistry were preformed. After grown on coverslips cells were fixed with ice-cold methanol. Non-specific protein-protein interactions were blocked with 5% BSA for 30 minutes at RT, then cells were incubated with the primary antibody overnight at 4°C. For secondary antibodies Alexa Fluor 488 green at a 1:200 dilution were used for 1h. Cells were counterstained with 4′-6′-diamidino-phenylindole (DAPI, Sigma, Part No.: D9542). All washing steps were preformed with PBS. Pictures were taken by a Nikon Eclipse E600 microscope with the help of Lucia Cytogenetics version 1.5.6 program. Details of antibodies and their appropriate dilutions are found in S3 Table.

### Western blot analysis

After cell lysis, protein concentration was measured by Bradford method. Twenty-five μg of denatured total protein were loaded onto a 10% polyacrylamide gel and were run for 30 min at 200 V. Proteins were transferred to PVDF membrane (Millipore, Billerica, MA, USA) for 1.5 h at 100 V. Ponceau staining was applied to determine blotting efficacy. Membranes were blocked with 3% w/v non-fat dry milk (Bio-Rad) in TBS for 1 h followed by incubation with the primary antibodies at 4°C overnight. After the washing steps (0.05 v/v% Tween-20 in TBS), appropriate secondary antibody was applied for 1 h, signals were detected by SuperSignal West Pico Chemiluminescent Substrate Kit (Pierce/Thermo Scientific, Part No.: 34080), and visualized by Kodak Image Station 4000MM Digital Imaging System. WB analyses were repeated 3 times. Ponceau staining was used to assess the equal loading of samples. Applied antibodies are indicated in S4 Table.

### Proliferation and migration assays of T3M4 pancreatic cancer cells

The effect of PSC-CCM on T3M4 pancreatic cells was assessed using a sulforhodamine-B (SRB) proliferation test. T3M4 cells were grown in 96 well plates (5000 cell/well) in 50% full-FBS RPMI with 50% conditioned PSC supernatant/no-FBS DMEM. Cells were fixed with 10 w/v% trichloroacetic acid at 0, 24, 48, 72 and 96 hours. After washing, cells were incubated with 0.4% sulphorodamine-B solution. Unbound SRB was eliminated with 1% acetic acid. Bound SRB was reconstituted with 10 mM TRIS buffer, absorbance was measured at 570 nm with microplate reader (LabsystemsMultiScan MS).

Migration of the T3M4 cells was examined using wound-healing assay. After plated in Petri dishes, cells were grown to full confluence. Proliferation was inhibited by with Mitomycin C, than the monolayer was wounded with a 100 μL pipette tip. After scratching cells were cultured with 50% RPMI+50% DMEM/PSC-CCM for 20h and photographed.

### Statistical analysis

Shapiro-Wilks test was used to assess normality. Two-tailed T-test with independent variables was used to compare means (normal distributions). ANOVA with Scheffe post-hoc test was used for multiple comparisons. Statistica (release 7) software was used for these calculations.

## Results

### Glucose transporters (types-1, -2, and -3) are expressed on human RLT-PSC and T3M4 cells

**Fig 1:**
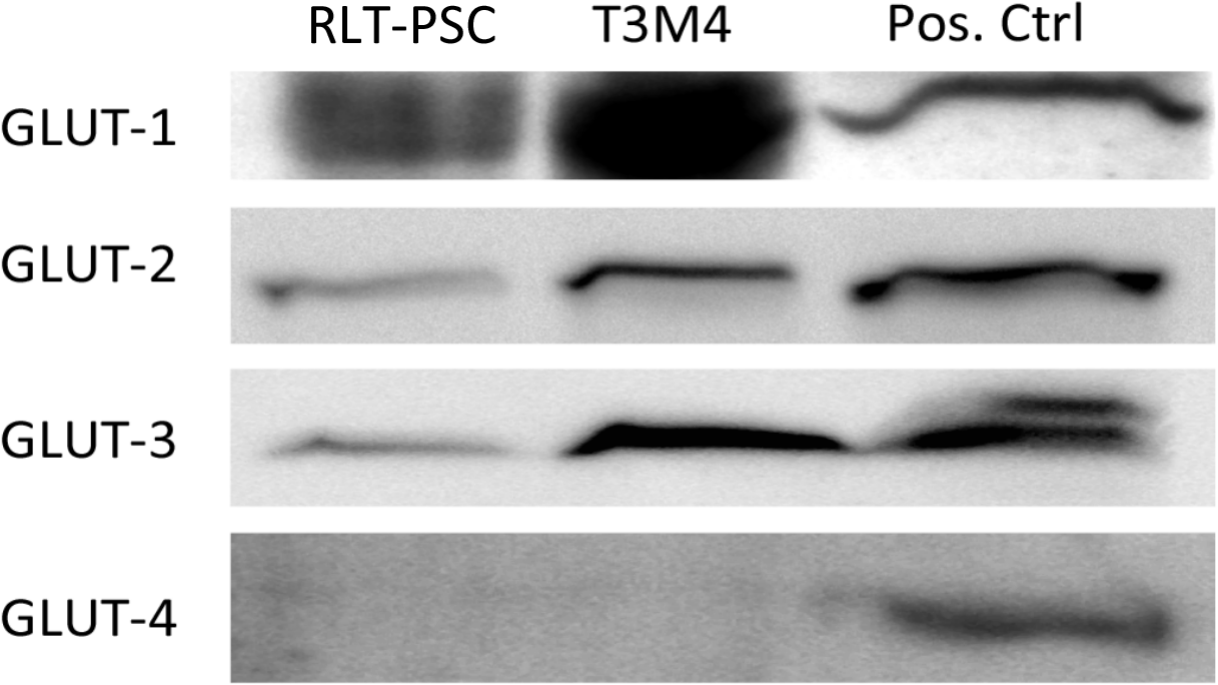
Western blot analysis of GLUT 1–4 on human RLT-PSC and T3M4 cells. GLUT-1, GLUT-2 and GLUT-3 are expressed on both human RLT-PSC and T3M4 PaC cells. GLUT-4 was neither detected on PSCs nor on T3M4 cells. Positive controls: hepatoma cell line HepG2 for GLUT-1 and GLUT-2, medulloblastoma DAOY for GLUT-3 and RD-40 rhabdomyosarcoma cells for GLUT-4.

Types-1, -2 and -3 GLUTs, but not GLUT-4 were detected in RLT-PSC and T3M4 PaC cell lysates by WB. (Fig 1).

### Chronic hyperglycemia and TGF-β1 induces a PSC activation

Increased α-SMA and type-1 collagen protein expression was detected by immunostaining after PSCs were exposed both to CHG and TGF-β1. Significantly higher type-1 and type-3 collagen protein levels were observed upon TGF-β1 treatment, both with (3.61-fold and 2.08-fold, p<0.05) and without (3.47-fold and 2.35-fold p<0.05) prior CHG exposure.

**Fig 2:**
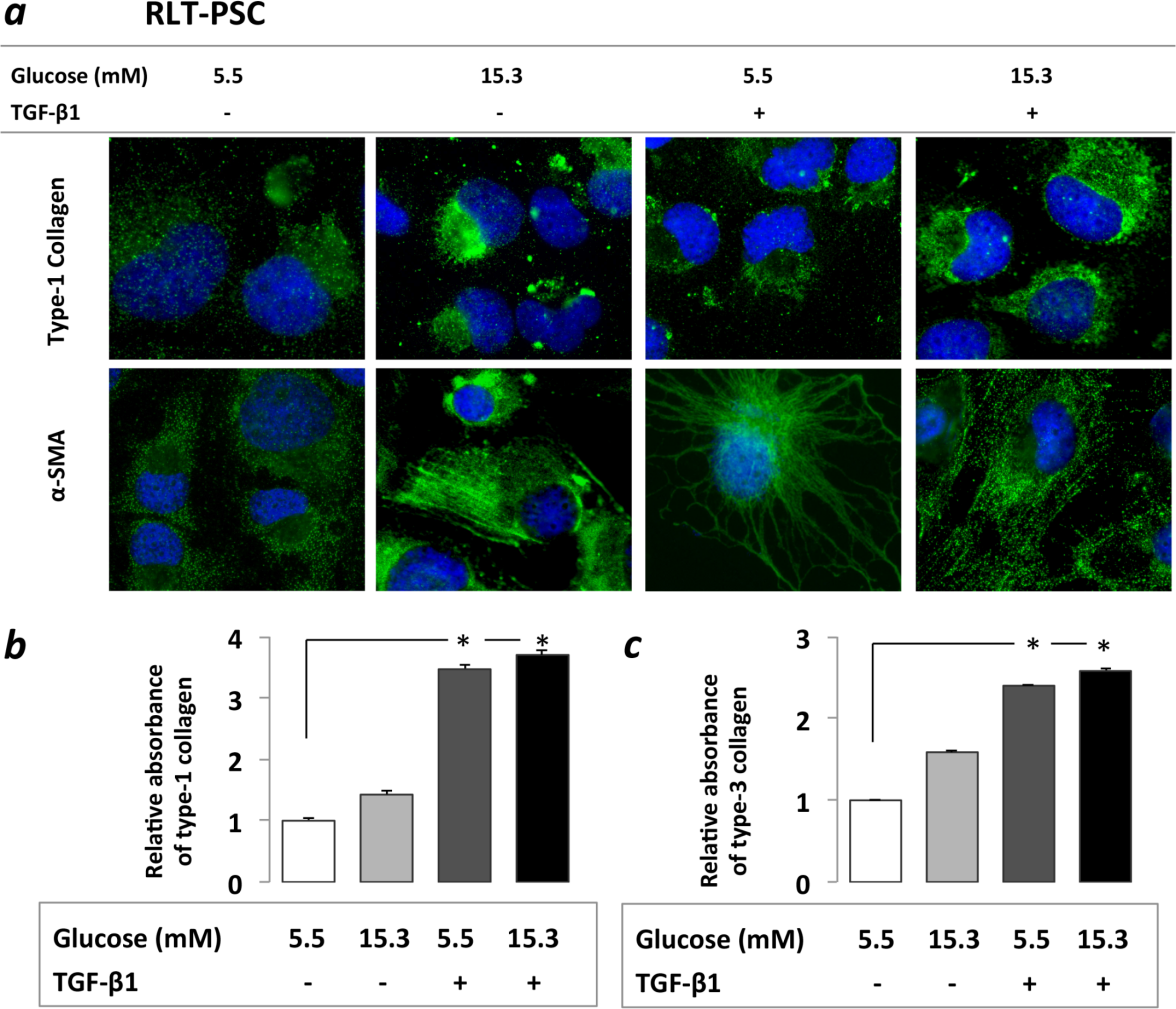
Immunocytochemistry *(a)* and ELISA assessment *(b)* of PSC activation. Increased α-SMA and type-1 collagen protein expression was found on immunocytochemistry after PSCs were exposed both to 21 days of hyperglycemia or 48h of TGF-β1 ***(a)*.** Increased type-1 and type-3 collagen levels were observed in PSC cell culture supernatant in the ELISA studies, however the increases were statistically only significant after the TGF-β1 treatments both with and without prior CHG exposure, but not after CHG exposure alone ***(b)*.** Significant differences (p<0.05) are indicated *

When PSCs were exposed only to CHG, a trend towards increased type-1 and -3 collagen productions (1.41-fold and 1.40-fold) was observed (Fig 2). These results suggest that chronic hyperglycemia may contribute to PSC activation and excessive ECM production.

### mRNA expression profiles in PSCs after treatments

Based on the results of the mRNA expression array we ranked the potentially altered pathways upon different treatments, and a set of differentially expressed genes was selected for further validation by real-time RT PCR (CXCL12, VCAN, FOS, LTBP2, COL5a1, THBS1, PPARγ, RND3, MMP1, DPP4). The biological plausibility in the view of the Metacore integrated pathway ranks served as a basis for selecting genes for further validation. All of the 10 selected genes displayed similar changes in real-time PCR validation as in the microarray.

Relative mRNA expression of CXCL12, FOS, LTBP2, THBS1 increased in PSCs after exposure to CHG, and the effect of TGF-β1 alone was limited. However, when we applied subsequently after CHG exposure, the TGF-β1 further augmented the up-regulation of CXCL12, LTBP2, THBS1 gene expressions. PPARγ, RND3, and MMP1 mRNA expression decreased after CHG exposure. Steady state level of VCAN and Col5a1 mRNA increased only after TGF-β1 treatment. Alterations gene expression at mRNA level in the human RLT-PSC cells after different treatments are indicated on S1 Fig.

### Increased CXCL12, IGFBP2 protein levels in RLT-PSC supernatants

**Fig 3:**
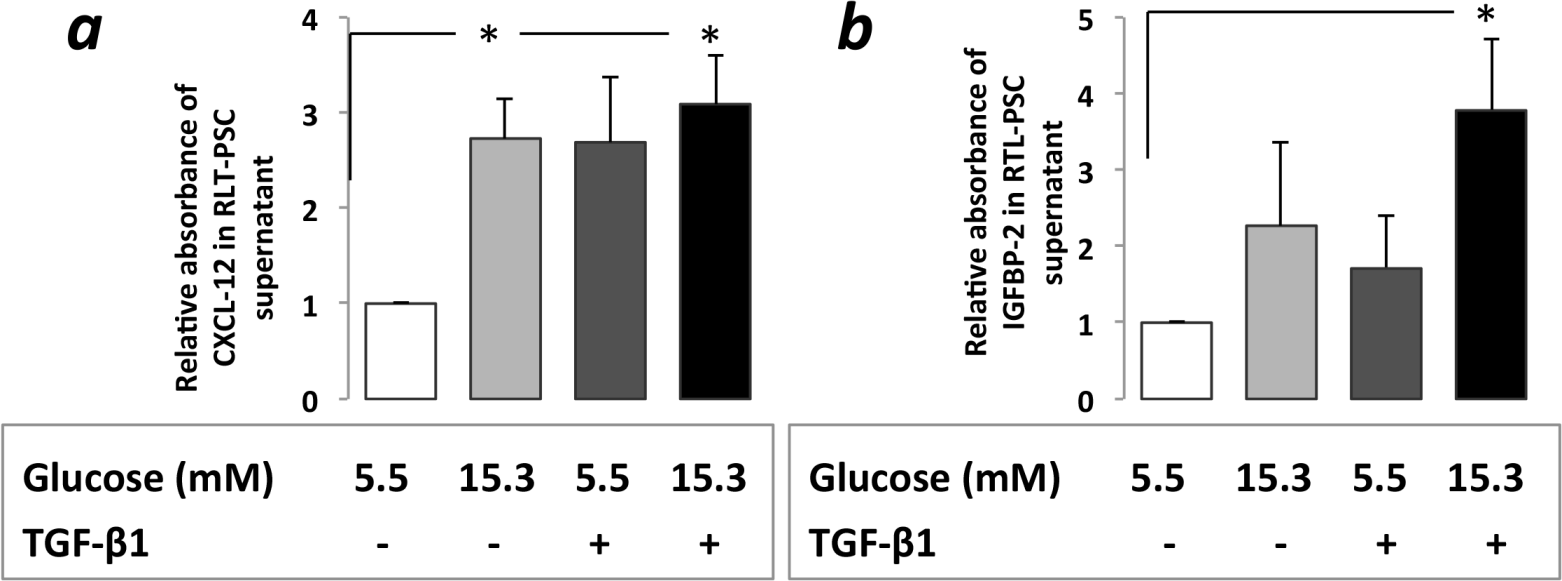
CXCL12 (*a*) and IGFBP2 (*b*) protein levels in RLT-PSC cell culture supernatant with ELISA assesment after exposure to hyperglycemia and/or subsequent TGF-β1 treatment. CXCL12 level was significantly increased in PSC cell culture after exposure to CHG while 48h TGF-β1 treatment with normal glucose concentration also resulted in more than 2-fold increase ***(a*)**. IGFBP2 protein level was increased in the cell culture medium after exposure to either CHG or TGF-β1. This alteration was only significant when PSCs were exposed to TGF-β1 subsequently after the CHG exposure. ***(b)*.** Significant differences (p<0.05) are indicated*

CXCL12 levels increased significantly in PSC supernatant after RLT-PSC cells were exposed to CHG regardless of subsequent TGF-β1 treatment (3.09-fold and 2.73-fold, p<0.05) (Fig 3A). Treatment of PSCs—kept previously in normal glucose concentration—with TGF-β1 for 48h resulted in a 2.69-fold increase of CXCL12 levels.

When the TGF-β1 treatment was applied subsequently after that PSCs were exposed to CHG it resulted in a significant (3.78 fold, p<0.05) IGFBP2 protein level elevation in the PSC supernatant (Fig 3B).

### Alteration of key signaling pathways in PSCs

**Fig 4:**
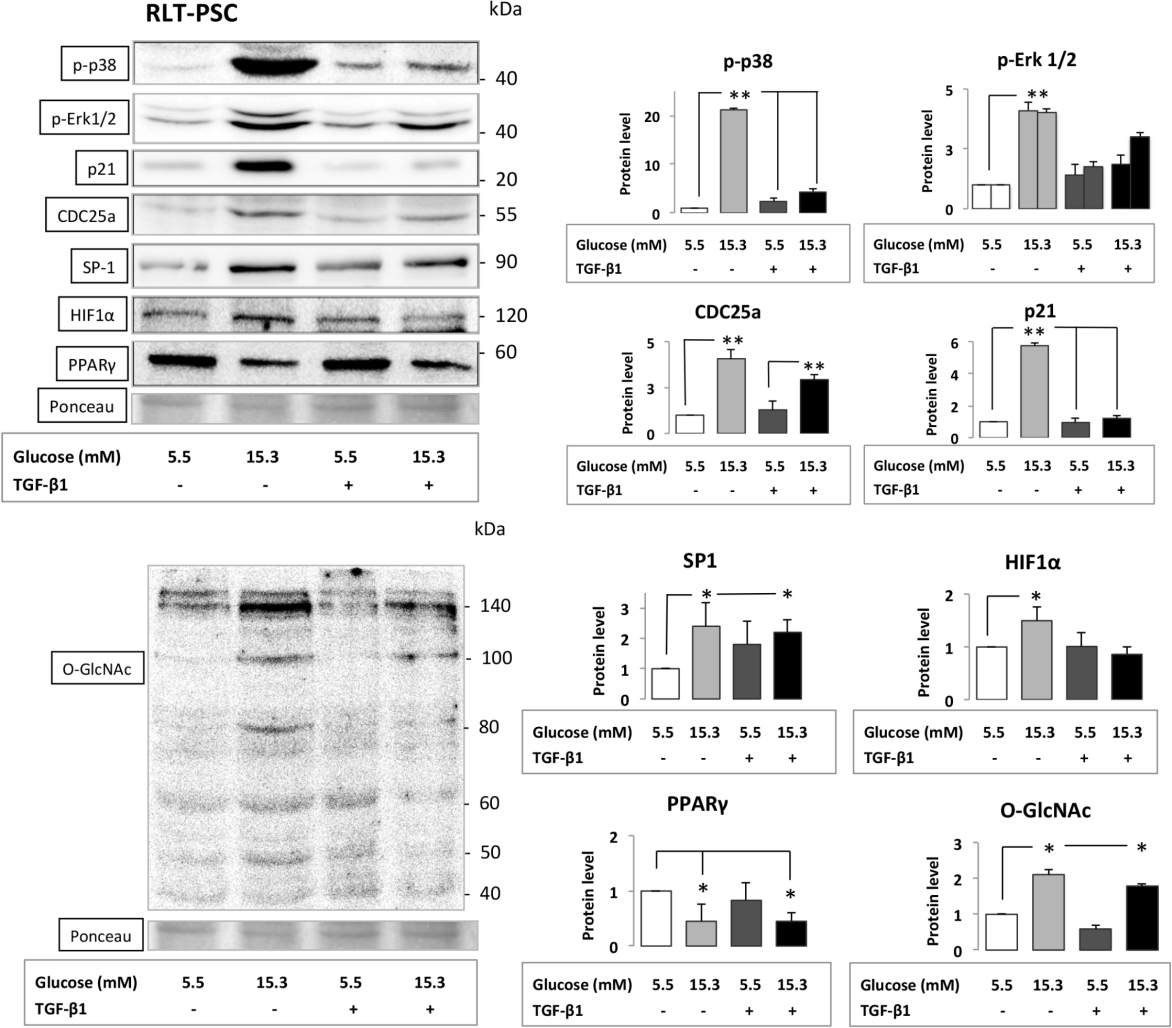
Series of Western blot experiments assessing key signaling molecule proteins from cell lysates of RLT-PSC cells. PSCs were exposed to CHG and/or to subsequent TGF-β1 treatment. Best representative images of key signaling molecules of the stellate cells are indicated in Western blot membranes. Ponceau staining was used to assess the equal loading of gels. Significant (p<0.05)* and highly significant differences (p<0.001)** are marked.

WB analysis of PSCs exposed to CHG both with and without subsequent TGF-β1 treatment demonstrated the most significant increase in the phosphorylation of ERK1/2 and p38 with subsequent induction of CDC25a and SP-1 proteins. The protein expressions of p21^waf1^, HIF1α, were also increased, while the amount of PPARγ decreased after PSCs were exposed to CHG (Fig 4). In addition, the hexosamine pathway was also induced as indicated by the altered β-O-linked N-acetylglucosamine (O-GlcNAc) protein modification patterns in PSCs exposed to CHG. WB results with the relative density values (significant and moderate alterations) are indicated on Fig 4. With the exception of marginal changes there were no conclusive, significant or major alterations in the amount of FAK, PTEN, AKT, PKC-α, c-FOS proteins in PSCs after different treatments. (S2 Fig)

### Increased Proliferation and Migration of T3M4 cells and the effect of a CXCR4 inhibitor

**Fig 5:**
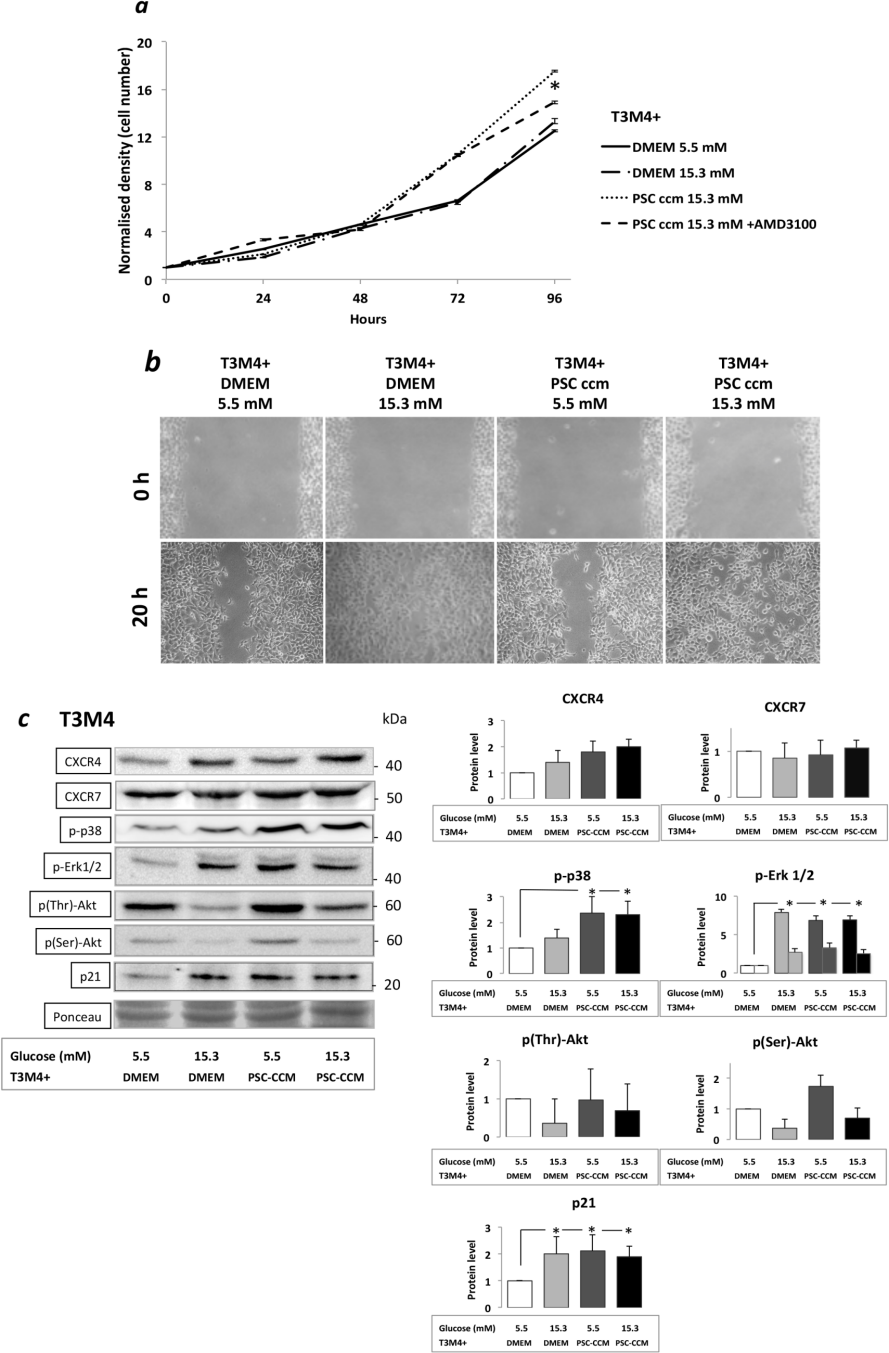
Proliferation and migration assays of T3M4 cells and the Western blot analyses of key signaling molecules: *(a)* Sulphorodamine B test. Proliferation of T3M4 cells incubated with different conditioned PSC culture medium and the CXCR4 inhibitor AMD3100 after 24, 48, 72 and 96 hours of treatment. T3M4 cell proliferation (solid line; untreated control) was increased significantly after incubation with PSC supernatant (dotted line) provided that PSC were prior exposed to CHG. The presence of the CXCR4 inhibitor AMD3100 (dashed line) could partially antagonize this proliferation induction after 96h. ***(b)* Migration of T3M4 cancer cells in a wound-healing assay.** Substantial effect of hyperglycemia on the migration of cancer cells: a rapid direct effect of elevated glucose level was found. There was no striking effect of conditioned PSC culture mediums on T3M4 cell migration. ***(c)* Western blot analyses of key signaling molecules in T3M4 cells.** Cancer cells were exposed to hyperglycemia or different conditioned PSC culture medium. Best representative images of key signaling molecules of the cancer cells are indicated in WB membranes. Ponceau staining was used to assess the equal loading of gels. Significant (p<0.05) differences are indicated.*

The proliferation of T3M4 cells significantly increased after 48h cultivation with CHG exposed PSC/CCM showing almost two fold increase (10.48) when compared to other treatments (DMEM-5.5: 6.58¸DMEM-15.3: 6.43, p<0.05) at 72h lasting throughout the experiment (96h). This proliferation promoting effect could be partially inhibited using the CXCR4 inhibitor, AMD3100 co-treatment (14.91 vs 17.54, p<0.05), but only after 96h of treatment (Fig 5A).

Migration of T3M4 tumor cells was assessed in a wound-healing assay. Both the exposure of cancer cells to direct hyperglycemia (cancer cells incubated in 50% DMEM-high glucose and 50% RPMI in a glucose concentation of 13.2mM) and the exposure to PSC CCM-5.5 enhanced migration after 20 hours compared to control cells. The most pronounced migration promoting effect was detected when cancer cells were incubated with conditioned PCS medium with high glucose content (CCM-15.3). (Fig 5B)

### Alteration of key signaling pathways in the T3M4 cells

In T3M4 cells, a massive (more than 5-fold) increase in phosphorylated ERK1/2 in parallel with a significant increase in p21 protein production were found after exposures both to hyperglycemia and to the PSC-CCM, despite that the T3M4 is a KRAS-Wild type ductal PaC cell line. Significant elevation in p-p38 was found in T3M4 cells exposed to both types of PSC-CCMs. Hyperglycemia exposure decreased the amount of p(Thr)Akt and p(Ser)Akt in T3M4 cells, in contrast, the exposure of cancer cells to PSC-CCM increased the amount of p(Ser)Akt (Fig 5C). Both receptors of CXCL12, the CXCR4 and the CXCR7 were expressed on the T3M4 cells (Fig 5C). T3M4 cells exposed directly to hyperglycemia or to different conditioned PSC culture media expressed increased amount of CXCR4, mostly after treatment of T3M4 cells with the culture medium of PSCs that were previously exposed to CHG. In contrast, we found no alteration in the CXCR7 protein expression on T3M4 cells after different treatments. WB results with the relative density values are indicated on Fig 5C. Only a modest, non-significant increase in c-FOS and in FAK proteins were found in T3M4 cells exposed to both types of PSC-CCMs nor the protein expressions of PTEN and PKCα were significantly altered in the T3M4 cells after different treatments. (S3 Fig)

## Discussion

The epidemiologic link between DM and PaC [1–3, 6] motivated this line of research. We hypothesized that CHG might induce trans-differentiation (activation) of PSCs and contribute to the development and progression of pancreatic fibrosis and cancer [15–18, 23]. The hypothesis was assessed using human, immortalized RLT-PSC cell line [13] in a unique experimental setup. A variety of PSC activating factors (e.g.: EtOH, TGF-β1, PDGF, TNFα, interleukines [IL-1,-6,-13], ROS) were previously identified, but the role of chronic (i.e.>14 days) hyperglycemia in PSC activation has not been investigated to date.

Previous studies reported a hyperglycemia induced activation of rat PSCs, but only up to 72 hours and without a genome-wide approach.[24–26] We found that human PSCs increased the cytoplasmic αSMA micro-filament formation and tended to increase major ECM protein constituents (type-1 and -3 collagens) upon exposure to CHG.

The genome-wide microarray approach was used to assess the glucose-induced alterations in mRNA expression patterns. The MetaCore pathway database software was used for preliminary identification of diabetes associated pathways, after the selection of the top 300 differentially expressed genes. Each molecular pathway was the result of the integration of multiple sets of genes with altered mRNA expressions in the microarray (e.g.: CXCL12 was identified as an integration of 12 genes with significantly altered mRNA expressions into a single signaling cascade). Subsequently we validated the mRNA expressions for a selected set of genes using real-time PCR. Key signaling molecules were then assessed at protein level in PSCs.

The phosphorylation of p38 was the most significant change in PSCs after exposure to CHG. We could not provide a clear-cut upstream pathway for hyperglycemia induced increased phosphorylation of p38 in PSCs, however similar results were reported in cultured human peritoneal mesothelial cells exposed to high glucose concentration, indicating that the p38 MAPK activation played a role in (the peritoneal) fibrosis development [27]. Activation of p38 pathway was also essential in the high glucose concentration induced epithelial-mesenchymal transition (EMT) of cultured human renal tubular epithelial cells [28] and EMT of acini in the pancreas may contribute to the ultimate PSC population [29]. In addition, the p38 pathway is activated during gluco-lipotoxicity induced apoptosis in insulin secreting INS-1 cells [30].

**Fig 6:**
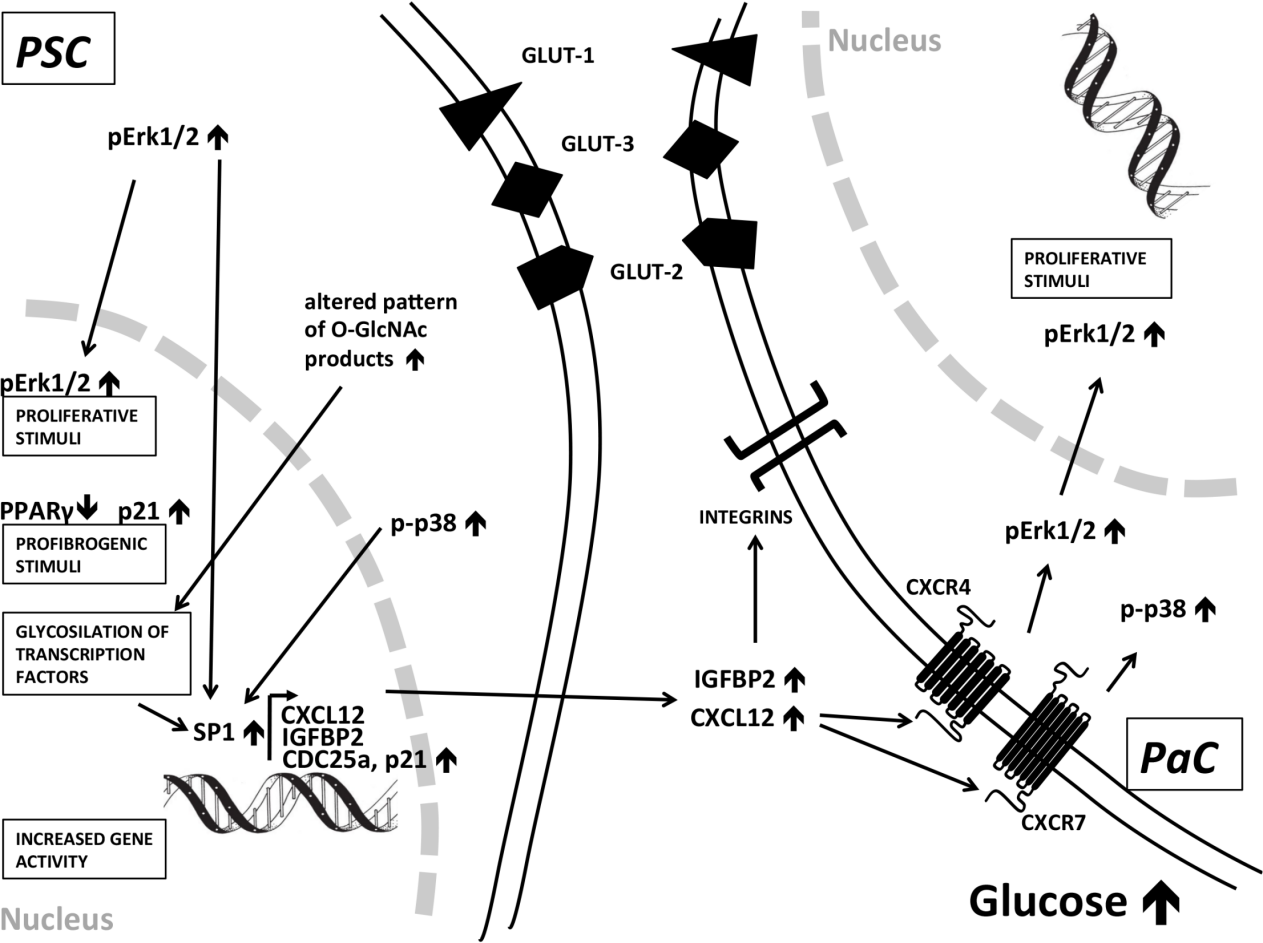
Summary of signaling pathways induced by chronic hyperglycemia in human pancreatic stellate cells and PaC cells. These results suggest a role of metabolic factors in PSC activation that may provide profibrogenic stimuli to a lesser extent proliferation signals in these stromal cells. Hyperglycemia also induced a cancer-associated secretion pattern of PSCs that may alter the pancreatic tumor microenvironment. Increased p38 phosphorylation may play an important role and CDC25/SP1 may convey the signals in the PSC nucleus that results in increased secretion of CXCL12 and IGFBP2 that may have an effect on pancreatic cancer cells.

SP1 was selected for WB analysis due to that the proximal promoter of CXCL12 is occupied by six putative SP1 binding motifs as confirmed by functional methods (in vitro mutagenesis) [31] and due to that SP1 transcription factor also binds to 200 bp upstream of the transcription initiation site of IGFBP2. SP1 also induces the transcription of p21 and CDC25 genes [32, 33]. We found a significant increase in SP1 protein amount in PSCs after exposure to CHG and this was independent from the effect of TGF-β1. SP1 overexpression in surgically resected human PaC was associated with aggressive disease and poor prognosis [34] and SP1 was computationally predicted to be the major regulator transcription factor in PaC [35]. We concluded that signaling via the hexosamine pathway, and the CHG induced increase of p-p38 and p-Erk1/2 coordinately all regulated the activation of SP1 that eventually resulted in the increased transcription of Sp1-dependent genes, including CXCL12, IGFBP2, CDC25a and p21 in PSCs (Fig 6) [36–39].

PPARγ agonists had inhibitory effect on hepatic stellate cell growth in a hepatic fibrosis model [40] and PPARγ ligand also inhibited the TGF-β-mediated lung myofibroblast differentiation [41] suggesting that downregulation of PPARγ may be a profibrogenic signal. Accordingly, the downregulation of PPARγ we first report here in human PSCs after CHG exposure may contribute to the other signals resulting in islet-specific pancreas fibrosis in diabetic patients[24]. Prior data suggested that progressive renal fibrosis was dependent on p21^WAF1^ expression [42] and the ECM expansion is a feature of diabetic nephropathy. Increased p21 protein expression reported here in PSC after CHG exposure may also be the consequence of the activation of SP1 binding to the p21 promoter region [32].

We found an altered pattern of glycosylated (O-linked N-acetylglucosamine) products in PSCs after exposure to CHG that may reflect the posttranslational modification of serine and threonine residues of transcription factors in the nucleus (Fig 4) [37]. The hexosamine pathway may also serve as a profibrogenic signal in PSCs such as in diabetic nephropathy. We concluded that chronic hyperglycemia may induce both profibrogenic stimuli (p-p38↑, p21↑, hexoseamine path↑, PPARγ↓), and proliferative signals (pERK1/2↑). In addition, the CHG exposure altered the secretion profile of PSCs, in part mimicking a cancer-associated PSC secretion profile (CXCL12↑, IGFBP2↑) and all of these three effects are likely to be evolved employing distinct intracellular pathways (Fig 6).

We first report that human PSCs express types 1, 2 and 3 glucose transporter proteins (GLUT-1, GLUT2 and GLUT-3).

Although previous reports related the CXCL12-CXCR4 axis to development, progression and recurrence of PaC [43–46], we first report that CHG is capable to increase the CXCL12 concentration in human PSC culture medium. Majority of PaC cell lines (co)express CXCR4 and CXCR7, the receptors of CXCL12.[43] Beta-arrestin-2 and K-Ras dependent pathways coordinate the transduction of CXCL12 signals,[43] and the source of CXCL12 in PaC tissue is the PSC that acts in a paracrine way on cancer cells. T3M4 cells express CXCR4 as the majority of other pancreatic cancer cell lines[43] and we also report that direct hyperglycemia and both types of PSC culture media exposures slightly increased the CXCR4 protein expression on T3M4 cells. In addition the partial inhibition of the CHG exposed PSC/CCM induced cancer cell proliferation by AMD3100, a specific CXCR4 blocker strongly refers to the functional role of the CXCL12-CXCR4 binding. The T3M4 cells also posessed CXCR7, a scavanger receptor for CXCL12. Despite that in our system some of the previously reported key beta-arrestin dependent signaling molecules (i.e.: CDK2 [47]) were not induced by the treatments, a role for the CXCR7 receptor induction of the MAPK pathways (i.e.: p38) in the T3M4 cells may still not be excluded.

We concluded that CHG influences the communication between PSCs and PaC cells and the increased CXCL12 levels may contribute to the progression of PaC. IGFBP2 overexpression was found in the pancreatic juice collected during ERCP in patients with PaC compared to those with benign pancreatic lesions [48]. We report that CHG exposure with subsequent TGF-β1 treatment significantly increases IGFBP2 protein production by PSCs. The increased IGFBP2 levels may not only be biomarkers of PaC, but also may contribute to the development and progression of PaC via their RGD domain binding to integrins (α5β1, α8β1, αv, αIIbβ3) [49].

The PSC-CCM medium significantly induced the proliferation of cancer cells in the SRB test after 72 hours, provided that PSCs were prior exposed to CHG. In addition to CXCR4-dependent mechanisms other signals are likely to contribute to the cancer cell proliferation induced by the PSC–CCM due to that AMD3100 could only partially antagonize this proliferation promoting effect.

Hyperglycemia profoundly increased the migration of T3M4 cells both directly and also indirectly via the alteration of PSC cell culture medium.

The GLUT-1 and GLUT-3 transporters that are expressed on T3M4 cells may indicate a potentially increased glucose uptake occuring concomittantly to the Warburg effect in cancer cells. It was reported that GLUT-1 is essential in the maintenance of self-renewal of the pancreatic cancer stem cell (CSC) population and the inhibition of GLUT-1 resulted in the inhibition of the tumor initiating capacity of CSC, suggesting that increased glucose metabolism is essential for maintaining stemness character of CSC [50].

Increased expression of p21 was described as an early event in pancreatic intraepithelial neoplasia (PanIN lesions). Overexpression of p21 was found to be progressive (normal duct-PanIN lesions-invasive carcinoma) [51]. Therefore the p21 increase reported here in T3M4 cancer cells and also in PSCs could be a thus far unrecognized effect of hyperglycemia. Conditioned PSC medium also increased p21 protein expression in K-Ras wild-type T3M4 PaC cells. We concluded that these stimuli could induce similar molecular changes that were previously found characteristic in early—PanIN—neoplastic lesions. We found that exposure of T3M4 cells both to hyperglycemia and to the PSC-CCM resulted in a striking increase in phosphorylation of ERK1/2 protein. These findings highlight the role of metabolic factors and the microenvironment in the activation of the ERK1/2 path even in a KRAS wild-type ductal PaC cell line.

## Conclusions

Our results suggest a role of metabolic factors in PSC activation vitiating the communication between PSCs and PaC cells. CHG induced a cancer-associated phenotype and secretion profile of PSCs that may alter the tumor microenvironment. This includes the induction of increased CXCL12 production by human pancreatic stellate cells and upregulation of CXCR4 expression on human cancer cells that in combination with the finding that hyperglycemia also directly enhanced cancer cell migration, suggests that it may promote both the tumor growth and the spread of pancreatic cancer. Furthermore, in the view that high glucose levels could induce molecular changes that are characteristic for PanIN lesions, this research may possibly refer to the role of metabolic factors in early events of cancer development.

## Supporting Information

S1 Fig Alterations in gene expressions in cells from the human RLT-PSC cell line after different treatments at mRNA level (microarray validation in real-time PCRs). (PDF)

S2 Fig Proteins produced without significant alterations in RLT-PSC cells after TGF-β1 and CHG treatment (Western blot, Ponceau staining for loading control). (PDF)

S3 Fig Proteins produced without significant alterations in T3M4 cells after incubation with PSC supernatant (Western blot, Ponceau staining for loading control). (PDF)

S1 Table Genes assessed in real-time PCR validation assays after the microarray experiment, with their major functions and TaqMan assay Ids used. (PDF)

S2 Table Antibodies used for measurements of type-1 and-3 collagen in ELISAssays. (PDF)

S3 Table Antibodies used in immunocytochemistry. (PDF)

S4 Table Antibodies used in the Western blot experiments. (PDF)
