# Supplementary material for: Chronic Hyperglycemia Induces Trans-Differentiation of Human Pancreatic Stellate Cells and Enhances the Malignant Molecular Communication with Human Pancreatic Cancer Cells
Source: PLoS One. 2015 May 26;10(5):e0128059. doi: 10.1371/journal.pone.0128059 (PMC4444240; doi:10.1371/journal.pone.0128059)
Supplement: S1 Fig — (PDF) [file pone.0128059.s001.pdf]

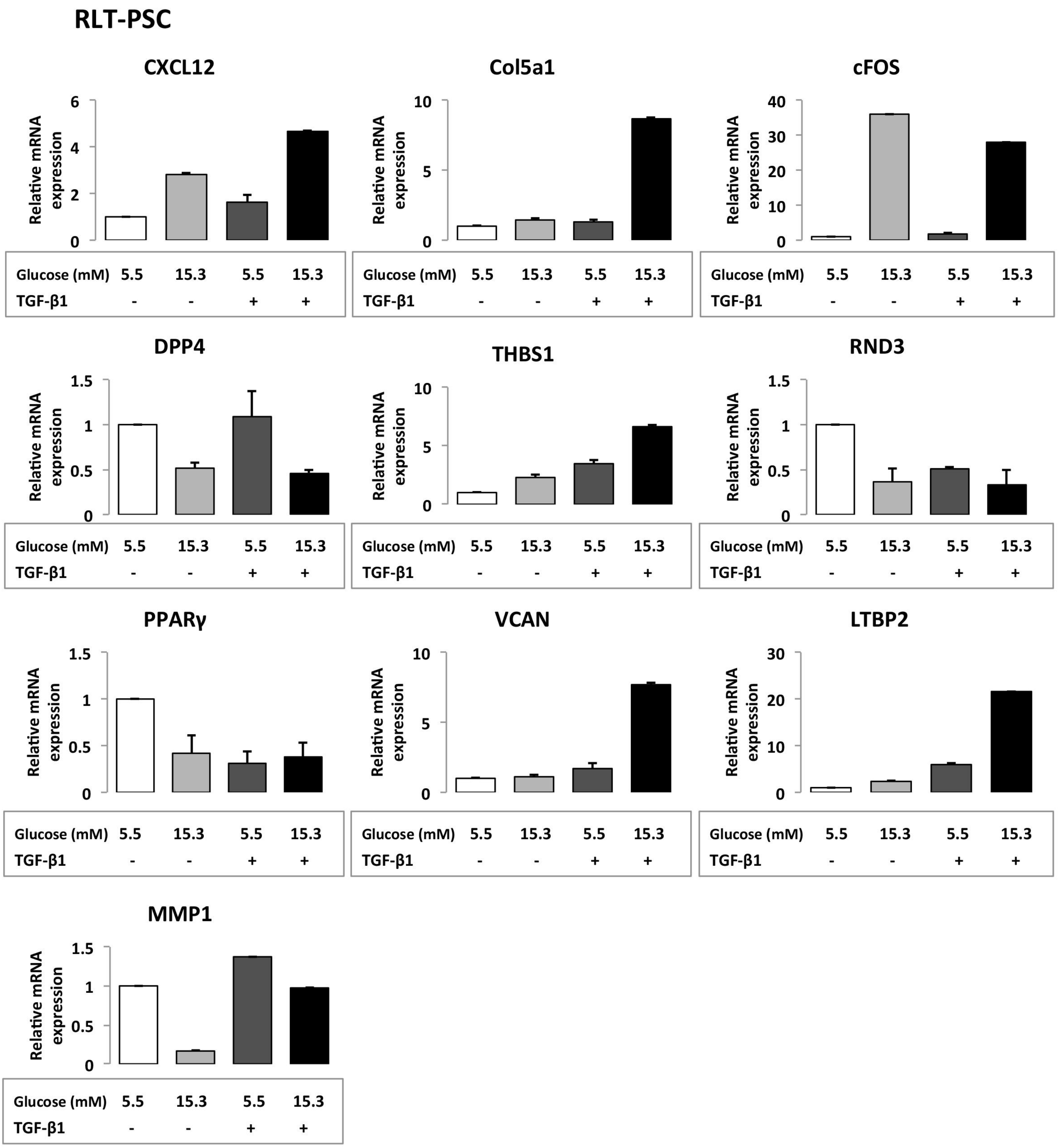
RLT-PSC
CXCL12
Relative mRNA expression
0
2
4
6
Glucose (mM) 5.5 15.3 5.5 15.3
TGF-β1 - - + +
Col5a1
Relative mRNA expression
0
5
10
Glucose (mM) 5.5 15.3 5.5 15.3
TGF-β1 - - + +
cFOS
Relative mRNA expression
0
10
20
30
40
Glucose (mM) 5.5 15.3 5.5 15.3
TGF-β1 - - + +
DPP4
Relative mRNA expression
0
0.5
1
1.5
Glucose (mM) 5.5 15.3 5.5 15.3
TGF-β1 - - + +
THBS1
Relative mRNA expression
0
5
10
Glucose (mM) 5.5 15.3 5.5 15.3
TGF-β1 - - + +
RND3
Relative mRNA expression
0
0.5
1
1.5
Glucose (mM) 5.5 15.3 5.5 15.3
TGF-β1 - - + +
PPARγ
Relative mRNA expression
0
0.5
1
1.5
Glucose (mM) 5.5 15.3 5.5 15.3
TGF-β1 - - + +
VCAN
Relative mRNA expression
0
5
10
Glucose (mM) 5.5 15.3 5.5 15.3
TGF-β1 - - + +
LTBP2
Relative mRNA expression
0
10
20
30
Glucose (mM) 5.5 15.3 5.5 15.3
TGF-β1 - - + +
MMP1
Relative mRNA expression
0
0.5
1
1.5
Glucose (mM) 5.5 15.3 5.5 15.3
TGF-β1 - - + +
