# Supplementary figures and images for: Chronic Hyperglycemia Induces Trans-Differentiation of Human Pancreatic Stellate Cells and Enhances the Malignant Molecular Communication with Human Pancreatic Cancer Cells
Source: PLoS One. 2015 May 26;10(5):e0128059. doi: 10.1371/journal.pone.0128059 (PMC4444240; doi:10.1371/journal.pone.0128059)

**RLT-PSC**

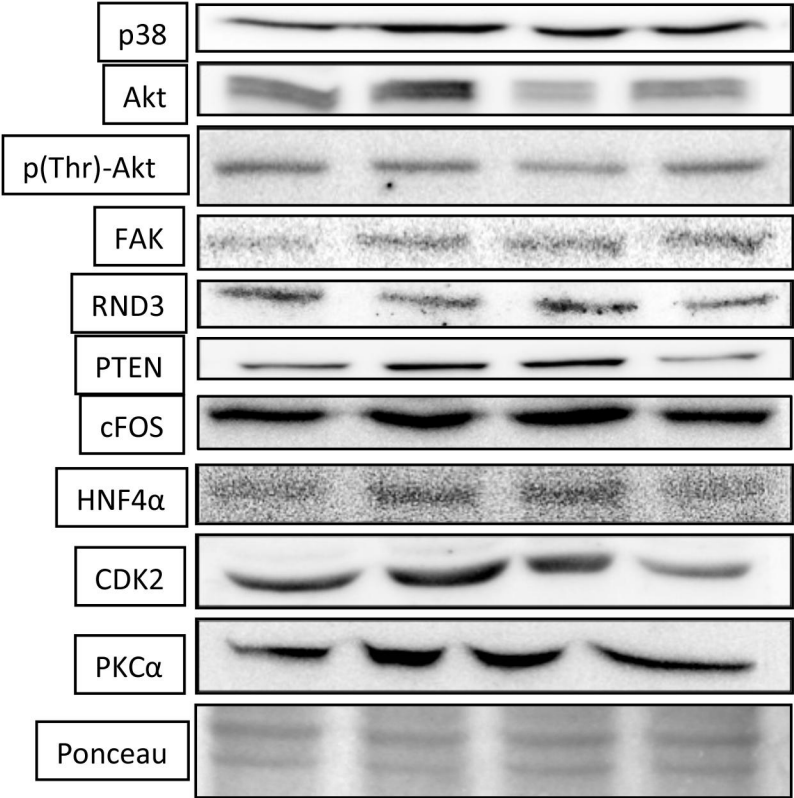

|              |     |      |     |      |
|--------------|-----|------|-----|------|
| Glucose (mM) | 5.5 | 15.3 | 5.5 | 15.3 |
| TGFβ         | -   | -    | +   | +    |

Supplement: S2 Fig — (PDF) [file pone.0128059.s002.pdf]

## T3M4

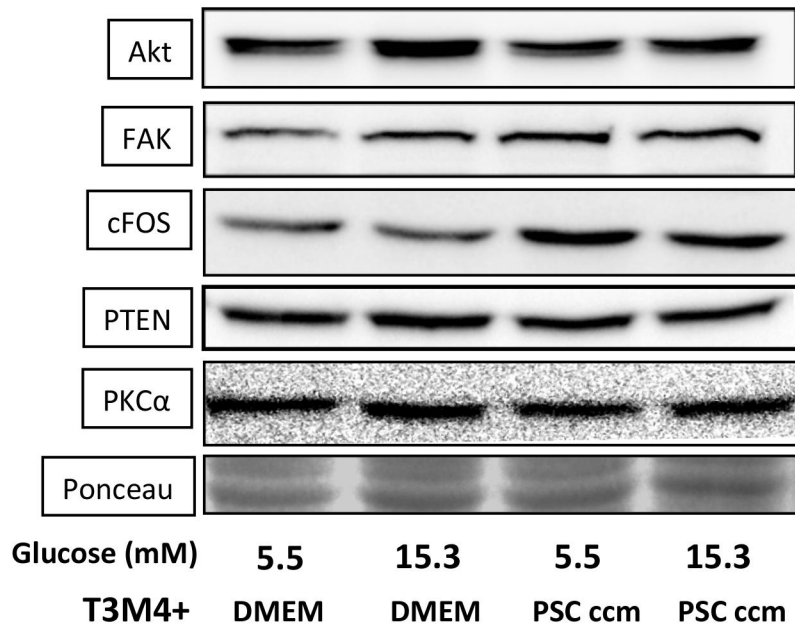

Supplement: S3 Fig — (PDF) [file pone.0128059.s003.pdf]
