# Supplementary material for: Chronic Hyperglycemia Induces Trans-Differentiation of Human Pancreatic Stellate Cells and Enhances the Malignant Molecular Communication with Human Pancreatic Cancer Cells
Source: PLoS One. 2015 May 26;10(5):e0128059. doi: 10.1371/journal.pone.0128059 (PMC4444240; doi:10.1371/journal.pone.0128059)
Supplement: S1 Table — (PDF) [file pone.0128059.s004.pdf]

Gene name abbreviation	Gene name	Gene function (as indicated in the NCBI/NLM/NIH)	Assay ID
CXCL12	Chemokine (C-X-C Motif) Ligand 12	ligand for the G-protein coupled receptor, chemokine (C-X-C motif) receptor 4	Hs00171022_m1
COL5A1	collagen, type V, alpha 1	regulate the assembly of heterotypic fibers	Hs00609088_m1
FOS	FBJ murine osteosarcoma viral oncogene homolog	forming the transcription factor complex AP-1 with proteins of the JUN family	Hs04194186_s1
DPP4	dipeptidyl-peptidase 4	serine exopeptidase	Hs00175210_m1
RND3	Rho family GTPase 3	negative regulator of cytoskeletal organization	Hs01003594_m1
PPARG	peroxisome proliferator-activated receptor gamma	regulator of adipocyte differentiation	Hs01115513_m1
VCAN	versican	role in cell adhesion, proliferation, migration and angiogenesis	Hs00171642_m1
LTBP2	latent transforming growth factor beta binding protein 2	member of the TGF-beta latent complex	Hs00166367_m1
MMP1	matrix metallopeptidase 1	unbild extracellular martix	Hs00899658_m1
THBS1	thrombospondin 1	adhesive glycoprotein that mediumtes cell-to-cell and cell-to-matrix interactions	Hs00962908_m1
