# Supplementary material for: Chronic Hyperglycemia Induces Trans-Differentiation of Human Pancreatic Stellate Cells and Enhances the Malignant Molecular Communication with Human Pancreatic Cancer Cells
Source: PLoS One. 2015 May 26;10(5):e0128059. doi: 10.1371/journal.pone.0128059 (PMC4444240; doi:10.1371/journal.pone.0128059)
Supplement: S2 Table — (PDF) [file pone.0128059.s005.pdf]

Antibody specificity	Clonality	Manufacturer	Cat No	Dilution used
Anti-Collagen I antibody	Rabbit Polyclonal	Abcam, UK	AB34710	1:1000
Anti-Collagen III antibody	Rabbit Polyclonal	Abcam, UK	AB 7778	1:1000
Anti-Rabbit Immunoglobulins/HRP	Goat polyclonal	DAKO	P0448	1:2000
