# Supplementary material for: Chronic Hyperglycemia Induces Trans-Differentiation of Human Pancreatic Stellate Cells and Enhances the Malignant Molecular Communication with Human Pancreatic Cancer Cells
Source: PLoS One. 2015 May 26;10(5):e0128059. doi: 10.1371/journal.pone.0128059 (PMC4444240; doi:10.1371/journal.pone.0128059)
Supplement: S3 Table — (PDF) [file pone.0128059.s006.pdf]

| **Antibody specificity** | **Clonality** | **Manufacturer** | **Cat No** | **Dilution used** |
|---|---|---|---|---|
| Anti-Collagen I antibody | Rabbit Polyclonal | Abcam, UK | AB34710 | 1:100 |
| Alpha-Actin (Smooth Muscle) (ACTA2) antibody | Monoclonal (E184) | Epitomics (AbCam) | 5264 | 1:1000 |
| Alexa Fluor 488 donkey anti-rabbit IgG (H+L) | Polyclonal | Invitrogene | A21206 | 1:200 |
