# Supplementary material for: Chronic Hyperglycemia Induces Trans-Differentiation of Human Pancreatic Stellate Cells and Enhances the Malignant Molecular Communication with Human Pancreatic Cancer Cells
Source: PLoS One. 2015 May 26;10(5):e0128059. doi: 10.1371/journal.pone.0128059 (PMC4444240; doi:10.1371/journal.pone.0128059)
Supplement: S4 Table — (PDF) [file pone.0128059.s007.pdf]

Antibody specificity	Clonality	Manufacturer	Cat No	Dilution used
**Anti-p38 antibody**	Rabbit Polyclonal	Cell Signaling	9212	1:100
**Anti-p(Thr180/Tyr182)-p38 antibody**	Rabbit monoclonal (D3F9)	Cell Signaling	4511	1:500
**Anti-p(Thr202/Tyr204)-p44/42 antibody**	Rabbit monoclonal (D13.14.4E)	Cell Signaling	4370	1:2000
**Anti-Akt (pan) antibody**	Monoclonal (C67E7)	Cell Signaling	4691	1:250
**Anti-p(Thr308)-Akt antibody**	Monoclonal (C31E5E)	Cell Signaling	2965	1:500
**Anti-p(Ser473)-Akt antibody**	Monoclonal (193H12)	Cell Signaling	4058	1:500
**Anti-FAK antibody**	Rabbit Polyclonal	Cell Signaling	3285	1:500
**Anti-RND3 antibody**	Mouse monoclonal (4)	Abcam, UK	AB50316	1:250
**Anti-PTEN antibody**	Rabbit monoclonal (D4.3)	Cell Signaling	9188	1:600
**Anti-cFOS antibody**	Rabbit Polyclonal	Abcam, UK	AB7963	1:400
**Anti-p21 antibody**	Rabbit Polyclonal	Abcam, UK	AB7960	1:1000
**Anti-PPARg antibody**	Rabbit Polyclonal	Abcam, UK	AB118521	1:200
**Anti-CDC25a antibody**	Mouse Monoclonal (DCS-120)	Santa Cruz	Sc-56264	1:200
**Anti-SP-1 antibody**	Rabbit Polyclonal	Cell Signaling	5931	1:1000
**Anti-HIF1a antibody**	Rabbit Polyclonal	Cell Signaling	3716	1:500
**Anti-HNF4a (G162) antibody**	Rabbit Polyclonal	Cell Signaling	3117	1:1000
**Anti-CDK2 antibody**	Rabbit Polyclonal	Sigma	C5223	1:1000
**Anti-PKCa antibody**	Rabbit Polyclonal	Cell Signaling	2056	1:750
**Anti-O-GlcNAc antibody**	Mouse monoclonal (CTD110.6)	Cell Signaling	9875	1:1000
**Anti-Glucose Transporter GLUT1 antibody**	Rabbit Polyclonal	Abcam, UK	AB652	1:500
**Anti-Glucose Transporter GLUT2 antibody**	Rabbit Polyclonal	Abcam, UK	AB54460	1:500
**Anti-Glucose Transporter GLUT3 antibody**	Rabbit Polyclonal	Abcam, UK	AB41525	1:2000
**Anti-Glucose Transporter GLUT4 antibody**	Rabbit Polyclonal	Abcam, UK	AB654	1:2000
**Anti-CXCR4 antibody**	Rabbit Polyclonal	Abcam, UK	AB2074	1:500
**Anti-CXCR7 antibody**	Goat polyclonal	Santa Cruz	Sc-107515	1:200
**Anti-Rabbit Immunoglobulins/HRP**	Goat polyclonal	DAKO	P0448	1:2000
**Anti-Goat Immunoglobulins/HRP**	Rabbit polyclonal	DAKO	P0449	1:2000
**Anti-Mouse Immunoglobulins/HRP**	Goat polyclonal	DAKO	P0447	1:2000
